# Prescription opioids among older adults: ten years of data across five countries

**DOI:** 10.1186/s12877-022-03125-0

**Published:** 2022-05-16

**Authors:** A. Hamina, A. E. Muller, T. Clausen, S. Skurtveit, M. Hesse, C. Tjagvad, B. Thylstrup, I. Odsbu, H. Zoega, H. L. Jónsdóttir, H. Taipale

**Affiliations:** 1 Norwegian Centre for Addiction Research (SERAF), Institute of Clinical Medicine, University of Oslo, PO Box 1171, 0218 Oslo, Norway; 2 School of Pharmacy, Faculty of Health Sciences, University of Eastern Finland, Kuopio, Finland; 3 Division of Reviews and Health Technology Assessments, Norwegian Institute of Public Health, Oslo, Norway; 4 Department of Mental Disorders, Division of Mental and Physical Health, the Norwegian Institute of Public Health, Oslo, Norway; 5 Center for Alcohol and Drug Research, Aarhus University, Aarhus, Denmark; 6 Centre for Big Data Research in Health, Faculty of Medicine & Health, UNSW Sydney, Sydney, Australia; 7 Centre of Public Health Sciences, Faculty of Medicine, University of Iceland, Reykjavik, Iceland; 8 Faculty of Psychology, School of Health Sciences, University of Iceland, Reykjavik, Iceland; 9 Department of Clinical Neuroscience, Karolinska Institutet, Stockholm, Sweden; 10 Niuvanniemi Hospital, Kuopio, Finland

**Keywords:** Opioids, Pharmacoepidemiology, Older adults, Nordic countries

## Abstract

**Abstract:**

**Background:**

Opioid use has increased globally in the recent decade. Although pain remains a significant problem among older adults, susceptibility to opioid-related harms highlights the importance of careful opioid therapy monitoring on individual and societal levels. We aimed to describe the trends of prescription opioid utilisation among residents aged ≥65 in all Nordic countries during 2009–2018.

**Methods:**

We conducted cross-sectional measurements of opioid utilisation in 2009–2018 from nationwide registers of dispensed drugs in Denmark, Finland, Iceland, Norway, and Sweden. The measures included annual opioid prevalence, defined daily doses (DDDs) per 1000 inhabitants per day (DIDs), and morphine milligram equivalents (MMEs) per user per day.

**Results:**

From 2009 to 2018, an average of 808,584 of adults aged ≥65 used opioids yearly in all five countries; an average annual prevalence of 17.0%. During this time period, the prevalence decreased in Denmark, Norway, and Sweden due to declining codeine and/or tramadol use. Iceland had the highest opioid prevalence in 2009 (30.2%), increasing to 31.7% in 2018. In the same period, DIDs decreased in all five countries, and ranged from 28.3 in Finland to 58.5 in Denmark in 2009, and from 23.0 in Finland to 54.6 in Iceland in 2018. MMEs/user/day ranged from 4.4 in Iceland to 19.6 in Denmark in 2009, and from 4.6 in Iceland to 18.8 in Denmark in 2018. In Finland, Norway, and Sweden, MMEs/user/day increased from 2009 to 2018, mainly due to increasing oxycodone utilisation.

**Conclusions:**

The stable or decreasing opioid utilisation prevalence among a majority of older adults across the Nordic countries coincides with an increase in treatment intensity in 2009–2018. We found large cross-national differences despite similarities across the countries’ cultures and healthcare systems. For the aged population, national efforts should be placed on improving pain management and monitoring future trends of especially oxycodone utilisation.

**Supplementary Information:**

The online version contains supplementary material available at 10.1186/s12877-022-03125-0.

## Introduction

Pain is a common symptom among community-dwelling people aged 65 or older, and can have a major impact on function, quality of life, and risk of disability in later life [1–4]. Pharmacotherapy is one of the most frequently applied forms of pain management, as approximately half of older adults report daily or as-needed analgesic use [5, 6]. However, there have been many changes to how the notion and the role of analgesics in pain treatment is perceived in the last two decades, and many treatment guidelines now emphasise non-pharmacological treatment, especially for chronic non-cancer pain [7–9]. Although paracetamol is recommended as the first-line drug, opioids play an important and symbolic part in this field [4]. In severe cancer pain and end-of-life pain opioids hold a quintessential role; for moderate-to-severe pain they are recommended to be prescribed for carefully selected and monitored older patients [4, 10–12]. Overall, pain treatment guidelines emphasise individual evaluation and caution when prescribing opioids, as older adults are susceptible to opioid-related adverse effects and drug-drug interactions [4, 10, 13]. Opioids increase the risk for severe adverse events, such as delirium, falls, and fractures [14–16], but also to the development of addiction, misuse, and consequent mortality [17]. The North American opioid epidemic has also influenced prescribing guidelines in Europe, and prescribing opioids for people over 65 has moved under closer inspection recently [17–19]. In addition, the cardiovascular risks associated with non-steroidal anti-inflammatory drugs (NSAIDs) have changed the patterns of use of non-opioid analgesics, which may increase the need for opioid analgesia [20].

Due to the ageing populations of the five Nordic countries, Denmark, Finland, Iceland, Norway, and Sweden [21], there is a growing need for healthcare services, but also for updated and age-specific information on national healthcare utilisation. As for opioids, the Nordic market has previously been dominated by the use of codeine and tramadol, commonly described as weak opioids, that have required less stringent documentation compared to strong opioids [22]. However, to reduce the use of tramadol, it has recently been scheduled as a narcotic in both Sweden and Denmark [23, 24]. Opioid utilisation has been described for the overall population in some Nordic countries, but recent descriptions lack a full picture of the utilisation in the five countries [22, 25–27]. Moreover, little is known about the patterns of opioid use in the older population. Age-specific studies are sorely needed, not only because opioids are most frequently used among older adults [26], but also due to the special needs in pain treatment and the age-related adverse effects associated with opioids [4]. Further, studies on whether the changing landscape of the analgesic market has impacted the use of opioids among older adults in the Nordic region are lacking.

The Nordic countries share history, cultural values, and political systems, and their healthcare systems are organized similarly and all register information on healthcare utilisation on a national level [28]. This makes the Nordic countries ideal for cross-country comparisons. In this study, we aimed to describe opioid utilisation trends and to compare utilisation patterns across countries among Nordic residents aged ≥65 years from 2009 to 2018. Specifically, we aimed to examine gender- and age group-related differences, and to compare utilisation patterns across countries.

## Material and methods

### Setting

All Nordic countries offer universal, tax-financed healthcare for their residents. This includes full or partial reimbursement of prescription drugs that are dispensed through community pharmacies. In all five countries, data on dispensed prescription drugs are electronically collected to nationwide and comprehensive, so-called prescription registers [28, 29]. With the exception of Iceland, the data from these registers are also aggregated into national drug consumption databases that are publicly available with a varied level of detail and maintained by the national authorities and register holders. In all countries except Finland, the prescription register data also contains data on non-reimbursed drugs. In this study, we used data from the drug consumption databases in Denmark, Finland, and Norway, and aggregated individual-level data from the prescription registers in Iceland and Sweden (Supplementary Fig. 1).

### Data sources

Each resident of a Nordic country has a unique personal identification number (PIN), which can be used to identify dispensations made to the resident over a lifetime. The data in the prescription registers include patient-centered information, such as age and gender, and drug-centered information, such as dispensation date, amount dispensed, and the Anatomical Therapeutic Chemical (ATC) code, which identifies the drug [28, 29]. The dispensed amount is included as defined daily doses (DDDs), which is the amount of assumed average maintenance doses per day when a drug is used for its main indication in adults, as defined by the World Health Organization (WHO) Collaborating Centre for Drug Statistics Methodology [30].

The prescription registers consist of data on drugs that have been dispensed from community pharmacies. Drugs administered in hospitals or other institutions that provide pharmacotherapy, are therefore not included in the register. Similarly, data on drugs sold over the counter (OTC) are not available at an individual level. During the study period, low-dose codeine in Denmark was the only analgesic opioid available without prescription in the Nordic countries.

In addition to the prescription registers, all Nordic countries have national statistical bureaus that offer data on the population demographics for different years [31–35]. We used these demographic data as denominators to calculate opioid utilisation prevalences.

### Opioids of interest

Opioids in this study were defined according to the ATC code N02A, i.e., analgesic opioids [30]. This means we did not include opioids for opioid maintenance therapy (N07BC) or antitussives (R05DA) in the analyses. Due to its apparent impact on opioid utilisation in the Nordic countries previously [22], we refer to the crude division of opioids by their potency based on their place on the WHO’s pain ladder [36], where “weak opioids” on the Nordic market include codeine, tramadol, and dextropropoxyphene, and “strong opioids” all other opioids. A list of the opioids available on the Nordic market and consequently included in this study is presented in Supplementary Table 1.

For Iceland and Sweden, we extracted individual-level data from the prescription registers on opioid dispensations among residents aged 65 or older at the time of the dispensation. These individual-level data were then aggregated into opioid utilisation statistics at gender and five-year age group level each year from 2009 to 2017 (Sweden) or 2018 (Iceland), depending on data availability.

For Denmark, Finland, and Norway, we extracted the data from the national drug utilisation databases [37–40]. Due to limitations in the level of detail from the publicly available data, the register holder in Finland (Kela) and a researcher from the Norwegian Institute of Public Health with access to closer detail (SS) extracted the data needed for this study. The data from 2009 to 2018 from these three countries included numbers of users and DDDs per individual opioid agents each year, identified by the ATC codes. As oxycodone and tramadol possess two individual 5th level ATC codes, we did not sum the numbers of users for these products in Denmark or Finland, as individuals may use more than one product per year. In Norway, these users were counted only once per year.

### Statistical analyses

We defined opioid utilisation prevalence as the number of individuals who had at least one opioid dispensation during 1 year divided by 100 inhabitants. The population numbers were extracted from the statistical bureau of each country. To estimate the amounts of opioids used in proportion to population size, we calculated the amounts of DDDs used by 1000 inhabitants per day, i.e., DDDs/1000 inhabitants/365 days (DIDs).

Next, we converted the utilized amounts of DDDs to oral morphine milligram equivalents (MMEs). This analysis was undertaken as DDDs may not always be the most clinically relevant metric to reflect amounts of opioid utilisation due to different opioid agents exhibiting different binding affinities to opioid receptors, thus possessing varying clinical potencies [41]. The MMEs were calculated according to their DDD values [30] and conversion factors supplied by the Norwegian Health Economics Administration (Supplementary Table 1) [42]. As ATC codes do not imply the opioid’s administration form, we made the assumption that the opioids were consumed in their most common form (Supplementary Table 1) [37], similarly to previous studies [26, 43]. The intensity of the opioid treatment for individuals was then estimated by dividing the utilized MMEs by the number of opioid users per day, i.e., MMEs/user/365 days.

Due to using full population data, we do not report confidence intervals or significance testing meant for describing uncertainty when estimating population values from smaller samples. All analyses were conducted using R (R Foundation for Statistical Computing, Vienna, Austria. https://www.R-project.org/).

## Results

**Fig. 1:**
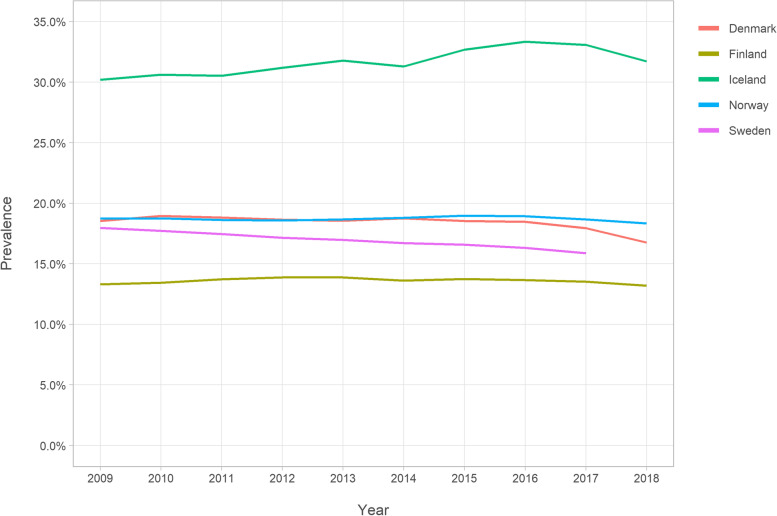
Opioid utilisation prevalence among Nordic residents aged ≥65

From 2009 to 2018, there were on average annually 808,584 opioid users among all persons aged ≥65 in all five countries combined, with an average annual opioid utilisation prevalence of 17.0%. Iceland had the highest prevalence of the five countries (Fig. 1 and Supplemental Table 2). The prevalence in Iceland ranged from 30.2% in 2009 to 33.4% in 2016, decreasing to 31.7% in 2018. The lowest prevalence was in Finland, ranging from 13.3% in 2009 to 13.9% in 2013, slightly decreasing to 13.2% in 2018. Opioid utilisation prevalence decreased somewhat during the observation periods in Denmark (from 18.6 to 16.8%), Norway (from 18.7 to 18.3%), and Sweden (from 18.0 to 15.9%).

**Fig. 2:**
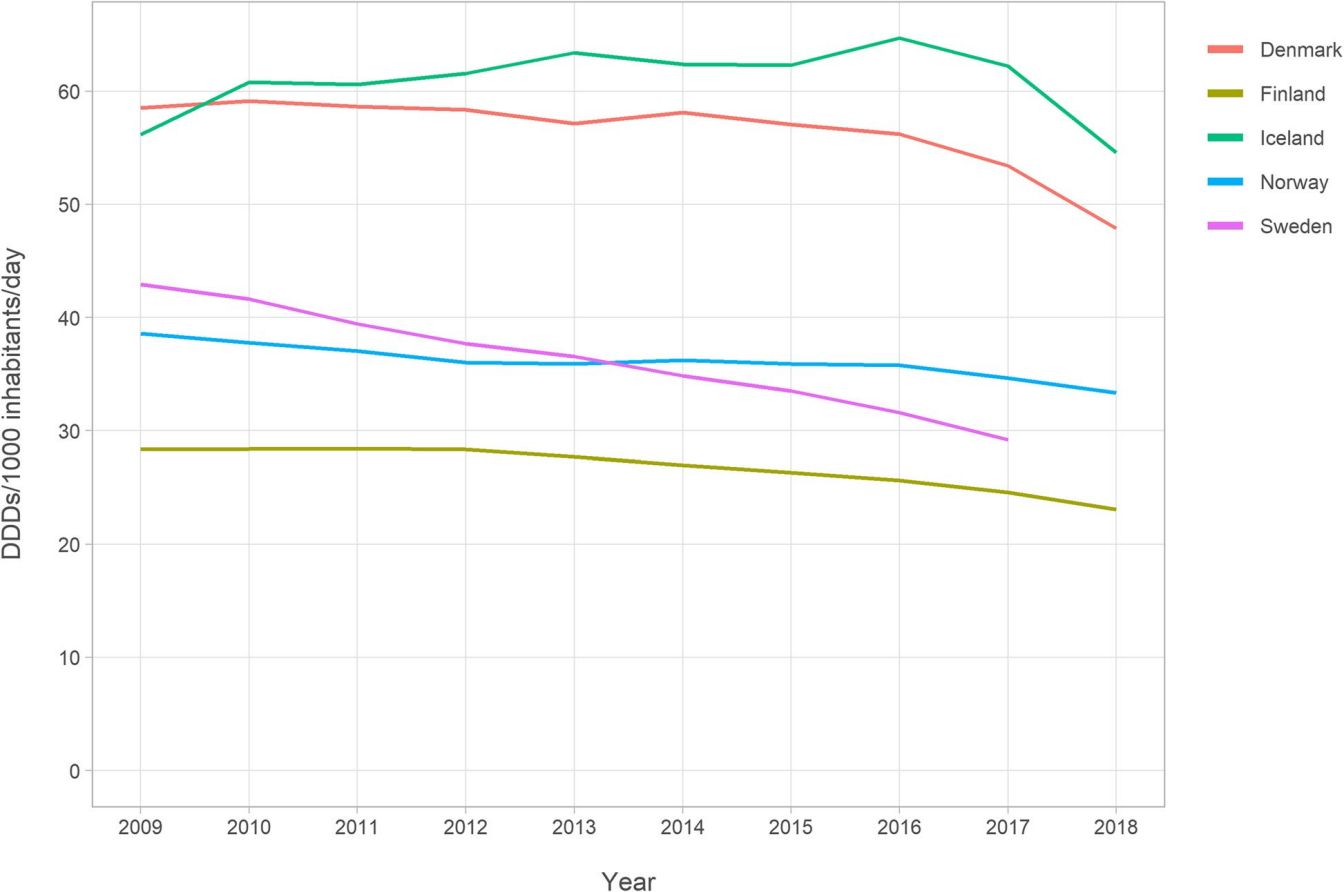
Opioid utilisation in Defined Daily Doses (DDD)s/1000 inhabitant/day among Nordic residents aged ≥65

Throughout the study period, opioid utilisation measured in DIDs was highest in Iceland, increasing from 56.1 in 2009 to 64.7 in 2016, and then decreasing to 54.6 in 2018 (Fig. 2 and Supplemental Table 3). This was followed by Denmark, where opioid utilisation decreased from 58.5 DIDs in 2009 to 47.9 DIDs in 2018. In Finland, DIDs decreased from 28.3 in 2009 to 23.0 in 2018, in Norway from 38.6 in 2009 to 33.3 in 2018, and in Sweden from 42.9 in 2009 to 29.2 in 2017.

**Fig. 3:**
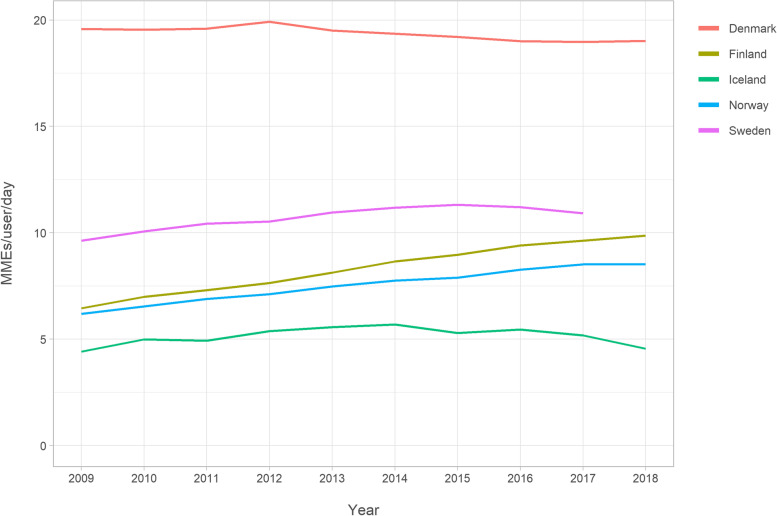
Opioid utilisation in morphine milligram equivalents (MME)s/user/day among Nordic residents aged ≥65

Measured in MMEs per user per day, older adults in Denmark utilized opioids most intensely during the entire study period (Fig. 3 and Supplemental Table 4), increasing from 19.6 in 2009 to 19.9 in 2012 and then decreasing to 19.0 in 2018. The least intense treatment was in Iceland, where intensity increased slightly from 4.4 MMEs/user/day in 2009 to 5.7 MMEs/user/day in 2014, and then decreased to 4.6 MMEs/user/day in 2018. In Finland, there was an increase from 6.5 in 2009 to 9.9 MMEs/user/day in 2018, in Norway from 6.2 in 2009 to 8.5 MMEs/user/day in 2018, and in Sweden from 9.6 in 2009 to 10.9 MMEs/user/day in 2018.

**Fig. 4:**
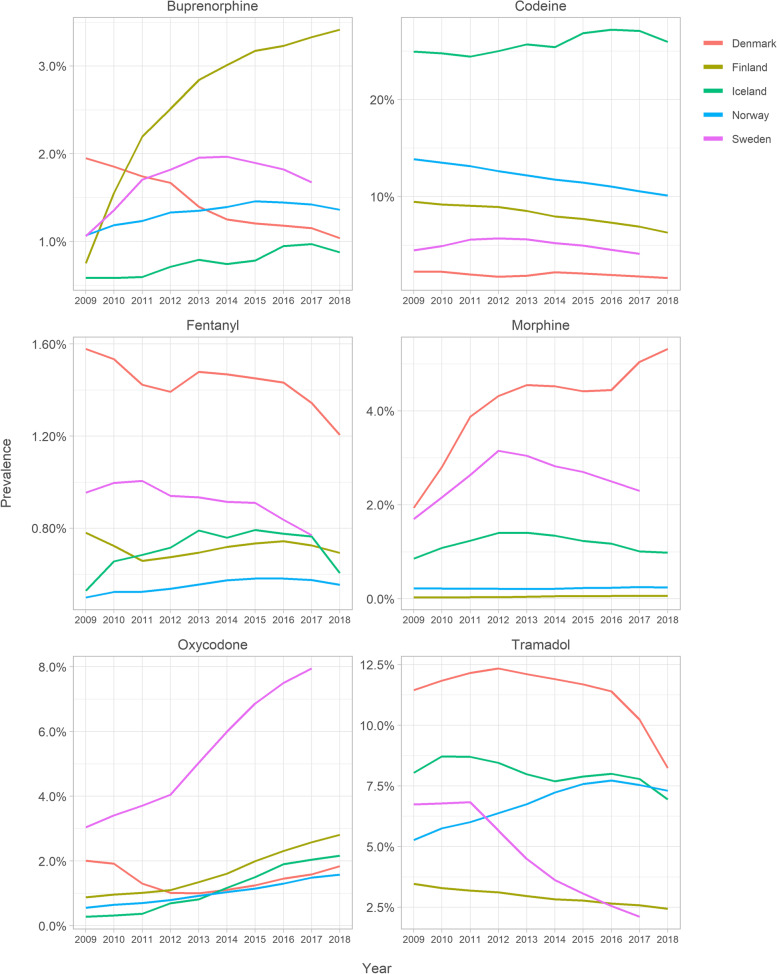
Prevalence of the most frequent opioids among Nordic residents aged ≥65. Oxycodone = N02AA05 in Denmark, Tramadol = N02AX02 in Finland. Prevalences of both N02AA55 and N02AJ13 remained < 0.01% during all years. Note scale change in the y-axes

The most frequently utilized opioid agents varied according to country (Fig. 4 and Supplemental Table 5). The weak opioids codeine and tramadol were the most common agents in all countries except Sweden, where oxycodone became the most frequent opioid in 2014, increasing in prevalence from 3.0% in 2009 to 7.9% in 2018. Oxycodone prevalence increased throughout the study period in the other countries, with the exception of Denmark, where it decreased from 2.0% in 2009 to 1.0% in 2012, then increasing to 1.9% in 2018. In Finland, codeine prevalence decreased from 9.5% in 2009 to 6.3% in 2018 and in Norway from 13.9% in 2009 to 10.1% in 2018. Tramadol prevalence decreased in Denmark from 11.5% in 2009 to 8.2% in 2018 and in Sweden from 6.7% in 2009 to 2.1% in 2017.

**Fig. 5:**
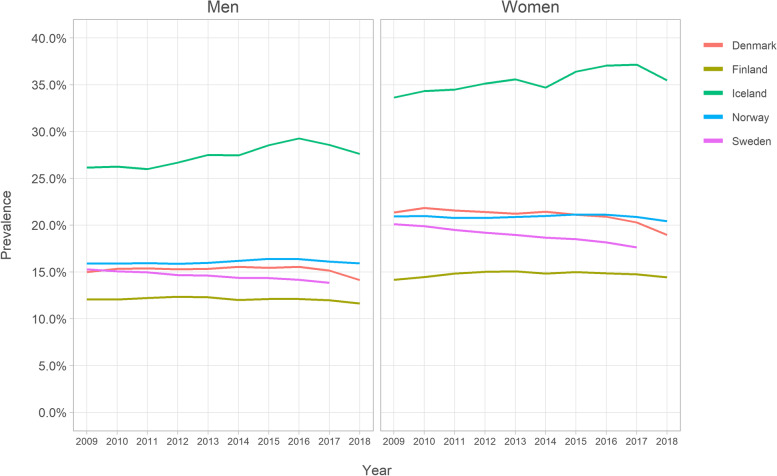
Prevalence of opioid utilisation according to gender

In all countries, women utilized opioids more frequently compared to men (Fig. 5 and Supplemental Table 6). In 2018, the prevalence of opioid utilisation among men in Iceland was 27.6 and 35.5% among women. Similarly, in 2018, the prevalence was 15.9% among men and 20.4% among women in Norway, 14.1% among men and 19.0% among women in Denmark, 11.6% among men and 14.4% among women in Finland, and, in 2017, 13.9% among men and 17.6% among women in Sweden. Of the individual opioid agents, buprenorphine displayed the most variation between men and women, with women’s utilisation prevalence being greater than the men’s (Supplemental Fig. 2). This difference was most visible in countries with the most buprenorphine utilisation, i.e., Finland and Sweden. Buprenorphine use prevalence was 2.1% among men and 4.5% among women in Finland in 2018, and 1.0% among men and 2.2% among women in Sweden in 2017.

**Fig. 6:**
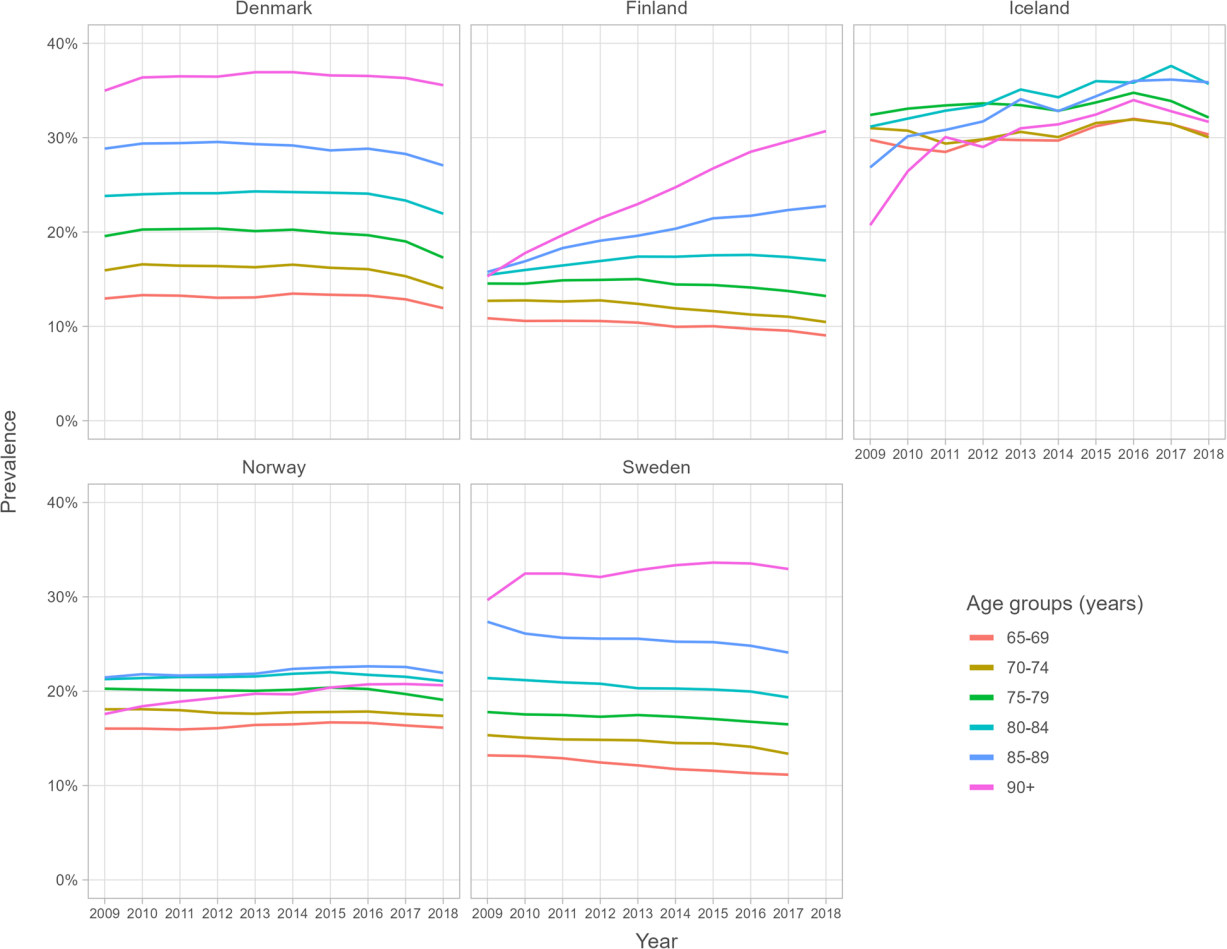
Prevalence of opioid utilisation according to age groups

There was large variation between the countries as to how much age influenced opioid utilisation (Fig. 6 and Supplemental Table 7). In Denmark and Sweden, the older age groups had higher prevalences of overall opioid utilisation throughout the study period compared to the younger age groups. In 2018, opioid prevalence among Danes aged 65 to 74 was 11.9%, but 35.5% among Danes aged 90 or more, and in 2017 it was 11.1% among Swedes aged 65 to 74, but 33.0% among Swedes aged 90 or more. In Finland, opioid prevalence decreased among those aged 65 to 69 from 10.9% in 2009 to 9.0% in 2018 but increased among the older age groups throughout the study period. In Iceland and Norway, there was little variation in opioid prevalence between the age groups.

## Discussion

To our knowledge, this is the first multinational study to describe opioid utilisation among community-dwelling older adults in the Nordic countries across several years. We found substantial variation in the opioid utilisation trends of the five Nordic countries among people aged 65 and over in 2009–2018. While older adults in Iceland had the highest prevalence, their intensity of treatment measured in MMEs per user was the lowest. With the exception of Iceland, the overall opioid utilisation prevalence remained stable or decreased during the study period. In Denmark, the overall intensity of the treatment was higher than in the other countries and remained stable, while the intensity increased in Finland, Norway, and Sweden.

Treating pain among older adults with opioids has been a topic of continuous discussion in the recent decades. This discussion has been guided by the opioid epidemic in Northern America, but also by the high prevalence of chronic non-malignant pain and the role of opioids, and the effects of pain in reducing function and quality of life in the older population [4, 10, 44, 45]. Given this background, our findings of relatively stable opioid utilisation prevalence among older adults in the Nordic countries are somewhat surprising, indicating that the discussion has had little impact on overall prescription patterns. However, these trends are similar to those discovered in previous studies on other adult populations from Nordic countries [23, 25–27, 43]. This indicates that the overall trends of opioid utilisation found in this study follow national patterns, rather than being age-specific variation. Since the early 2000s, codeine and tramadol have been the most commonly used opioid agents in all five countries [22]. It is likely that this preference for weak opioids has been influenced by requirements for additional documentation when prescribing strong opioids, which are classified as narcotics. Products containing tramadol were, however, rescheduled as narcotic in Sweden in 2012 [23] and in Denmark in 2017 [24], leading to a rapid reduction in tramadol utilisation in both countries [24]. In Iceland, the high prevalence and low intensity of opioid utilisation indicates that small amounts of especially codeine are being dispensed to a relatively high proportion of the older population. As the other countries are reducing codeine utilisation, the underlying causes of this prescribing disparity should be explored further.

Our findings on the steadily increasing intensity of opioid treatment for older adults in Finland, Norway, and Sweden is likely to be a result of the increase in the utilisation of especially oxycodone and the decrease in codeine and/or tramadol utilisation. The increase in oxycodone use has been especially significant in Sweden, where it has been replacing tramadol, codeine, and morphine, but also dextropropoxyphene, which was withdrawn from the market in 2011 [23]. Preferring low-dose oxycodone to weak opioids among older adults may be a viable strategy due to the adverse effects, drug-drug interactions, and genetic variation in the metabolism of tramadol and codeine [4]. However, due to the high potency of oxycodone and its addictive and misuse potential [46], its utilisation trends and the broader effects of these changes need to be followed up and critically assessed.

In Finland, part of the increase in MMEs per user can be attributed to increased buprenorphine use. Especially among older adults, the drug is almost solely used transdermally after the marketing launch of the long-acting patches in 2009 [47]. Interestingly, buprenorphine use appears to be very gendered in Finland and Sweden, with women using the drug far more frequently. This finding can partly be explained by older women reporting more frequent and intense pain compared with men of the same age [4, 48] and/or a lower threshold for seeking medical help for pain [49]. Further, the discrepancy may partly be related to the higher prevalence of dementia among women [50], which increases the likelihood of transdermal opioid use over oral ones [47, 51] possibly due to memory and ingestion problems, but also because of the perceived ease of use for caretakers. Moreover, the prevalence of illnesses that cause chronic pain, such as osteoporosis [52] and osteoarthritis [53] is higher among women, also contributing to higher prevalence of opioid utilisation among women across the Nordic countries. However, we cannot fully explain why specifically frequent buprenorphine use is strongly gender-related.

Our age-stratified results indicate that different strategies are used across the countries in the treatment of pain among the oldest people. In Denmark, Sweden, and in the later years in Finland, the oldest age groups have had a higher prevalence of opioid utilisation compared with younger age groups. While the prevalence of opioid utilisation among all Finnish persons over 65 remained stable throughout the study period, it increased significantly among Finns aged 85 or older. Although this implies a shift in the opioid prescribing culture for the oldest old, part of the increase may come from prioritizing home care and assisted housing over institutional care [54, 55], increasing the time people with higher disease burden purchase their medicines through community pharmacies [54]. Similarly to our results, a previous study found that morbidity in Finnish long-term care facilities has increased and opioid use almost tripled from 2003 to 2017 [56]. In contrast, we did not find large differences between the age groups in Iceland or Norway. Previously, increasing trends of strong opioid use have been reported among Norwegian older adults in 2005–2010 [57], and although we found that people aged 90 or older increased their overall opioid utilisation in Norway, the previous trends appear to have leveled off. Future studies need to examine the reasons for the differences in the opioid utilisation of the oldest age groups across the Nordic countries, and whether the treatment of moderate-to-severe pain is of similar quality regardless of these differences.

A major strength of this study is the nationwide coverage of the used registers. All Nordic prescription registers cover all filled prescriptions from community pharmacies, except for non-reimbursed drugs in Finland [29]. Therefore, the data are not biased by regionality or socioeconomic status. In Finland, opioids are widely reimbursed, but codeine effervescent tablets, products containing ibuprofen and codeine, and products containing oxycodone and naloxone were not covered during the study period. While the market share of effervescent codeine tablets is unknown, it is likely to be small. Products with codeine and ibuprofen made up approximately 2.2% of the country’s overall utilisation of codeine and products with oxycodone and naloxone 14.3% of the utilisation of oxycodone in 2018 [58]. Similarly, a limitation of this study is the incomplete data on drugs utilized in institutions and OTC drugs, i.e., codeine products in Denmark. Data on drugs utilized in long-term care may be incomplete especially in Finland and in Norway, where dispensations through community pharmacies in these institutions are more rare compared to Denmark, Iceland, and Sweden [28]. The opioid utilisation numbers for Finland and Norway may thus be underestimated. Similarly, part of the apparent increase in opioid prevalence among the oldest age groups in Iceland from 2009 to 2011 may be related to increased coverage of nursing homes in the data. As for other limitations, we did not gather data on non-pharmacological methods to reduce pain or on non-opioid analgesics, as this was a study on opioid utilisation. There are recent Nordic reports on both the utilisation of NSAIDs [20] and paracetamol [59]. Without data on individual factors, such as physical function and disease burden, we have not assessed the appropriateness of treatment in any way. An additional limitation in our analysis is the focus on national data, which is insufficient to describe region-specific variation, which is likely to exist between rural and urban areas.

## Conclusions

Stable or decreasing opioid utilisation prevalence trends among the majority of older Nordic residents coincides with increasing intensity of treatment in 2009–2018. While the decrease in codeine and tramadol utilisation may reduce the overall risk of adverse effects, prescribers’ clinical judgment needs to be applied to balance pain management, quality of life, and physical and mental functioning. National efforts should be placed on improving pain management among the older population and monitoring future trends of especially oxycodone utilisation. Our findings on age and gender-specific differences underline the importance of conducting stratified analyses in future drug utilisation studies.

## Supplementary Information

**Additional file 1: Supplemental Figure 1.** Data sources of the study. **Supplemental Figure 2.** Annual prevalence of buprenorphine utilisation according to gender among Nordic adults aged ≥65. **Supplemental Table 1.** Opioids on the Nordic market during 2009–2018. **Supplemental Table 2.** Values in Fig. 1: Annual prevalence (%) of opioid utilisation among Nordic residents aged ≥65. **Supplemental Table 3.** Values in Fig. 2: Opioid utilisation in Defined Daily Doses (DDD)s/1000 inhabitant/day. **Supplemental Table 4.** Values in Fig. 3: Opioid utilisation in morphine milligram equivalents (MME)s/user/day. **Supplemental Table 5.** Values in Fig. 4: Annual prevalence (%) of the most frequent opioids among Nordic residents aged ≥65. **Supplemental Table 6.** Values in Fig. 5: Annual prevalence (%) of opioid utilisation according to gender. **Supplemental Table 7.** Values in Fig. 6: Annual prevalence (%) of opioid utilisation according to age group. **Supplemental Table 8.** Values in Supplemental Figure 2: Annual prevalence (%) of buprenorphine according to gender.

## Data Availability

The drug utilisation databases and statistics bureau data is available on the internet, see references [31–35, 37, 39, 40].

## Abbreviations

ATC: Anatomical-therapeutic-chemical categorization
DDDs: Defined daily doses
DIDs: Defined daily doses per 1000 inhabitants per day
MMEs: Morphine milligram equivalents
NSAIDs: Non-steroidal anti-inflammatory drugs
OTC: Over-the-counter
PIN: Personal identification number
WHO: World health organization
