# Supplementary material for: Prescription opioids among older adults: ten years of data across five countries
Source: BMC Geriatr. 2022 May 16;22:429. doi: 10.1186/s12877-022-03125-0 (PMC9112605; doi:10.1186/s12877-022-03125-0)
Supplement: Supplementary file 1 — Additional file 1: Supplemental Figure 1. Data sources of the study. Supplemental Figure 2. Annual prevalence of buprenorphine utilisation according to gender among Nordic adults aged ≥65. Supplemental Table 1. Opioids on the Nordic market during 2009–2018. Supplemental Table 2. Values in Fig. 1: Annual prevalence (%) of opioid utilisation among Nordic residents aged ≥65. Supplemental Table 3. Values in Fig. 2: Opioid utilisation in Defined Daily Doses (DDD)s/1000 inhabitant/day. Supplemental Table 4. Values in Fig. 3: Opioid utilisation in morphine milligram equivalents (MME)s/user/day. Supplemental Table 5. Values in Fig. 4: Annual prevalence (%) of the most frequent opioids among Nordic residents aged ≥65. Supplemental Table 6. Values in Fig. 5: Annual prevalence (%) of opioid utilisation according to gender. Supplemental Table 7. Values in Fig. 6: Annual prevalence (%) of opioid utilisation according to age group. Supplemental Table 8. Values in Supplemental Figure 2: Annual prevalence (%) of buprenorphine according to gender. [file 12877_2022_3125_MOESM1_ESM.docx]

Supplemental Figure 1. Data sources of the study.


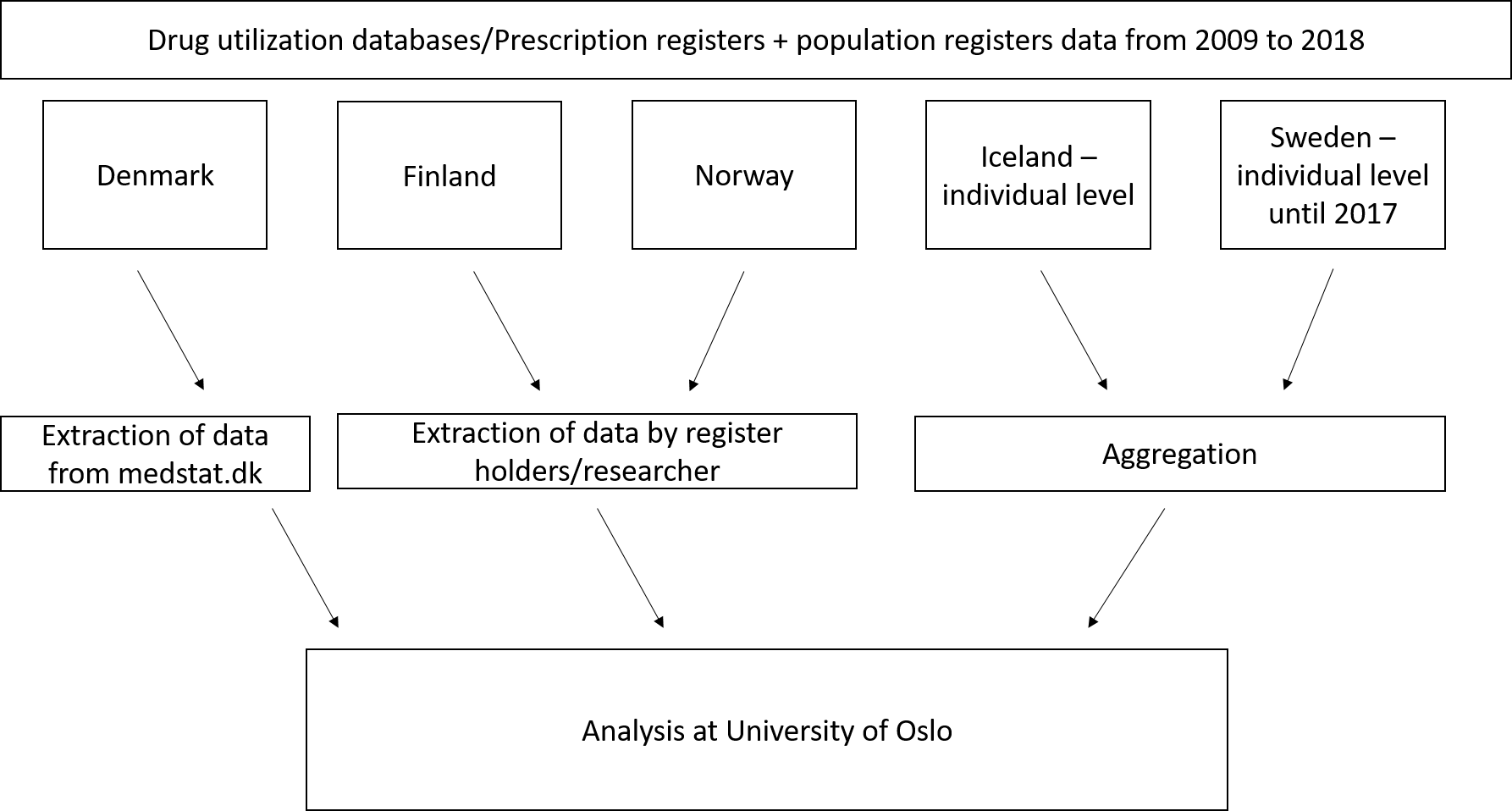


Supplemental Figure 2. Annual prevalence of buprenorphine utilisation according to gender among Nordic adults aged ≥65.

*
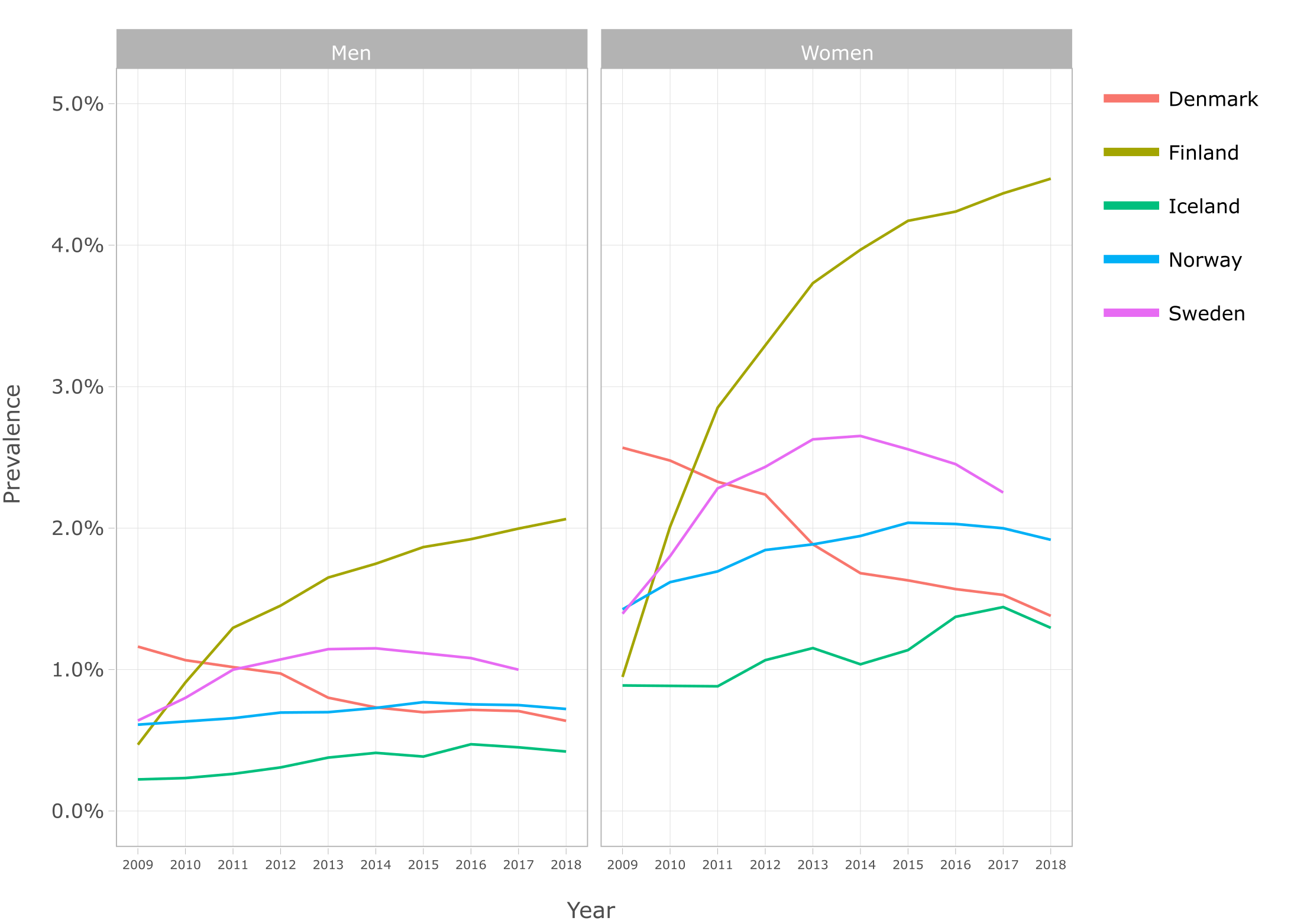
*

Supplemental Table 1. Opioids on the Nordic market during 2009-2018.

| **Opioid (ATC codes)**^1^ | **Most frequent form of administration**^2^ | **DDD (mg)**^1^ | **Conversion factor**^3^ | **Exceptions** |
| --- | --- | --- | --- | --- |
| **Weak opioids** |  |  |  |  |
| Codeine combinations (N02AJ06; N02AJ07; N02AJ in Denmark) | Oral | 30 (N02AJ07)  90 (N02AJ06) | 0.05 | Data on OTC products not available in Denmark;  Data on effervescent tablets and ibuprofen combinations not available in Finland |
| Dextropropoxyphene (N02AC04) | Oral | 200 (N02AC04)  70 (N02AC54) | 0.05 | Removed from the Nordic market in 2011 |
| Tramadol (N02AX02, N02AJ13) | Oral | 300 (N02AX02) 150 (N02AJ13) | 0.1 | Annual prevalence of N02AJ13 reported separately in Finland |
| **Strong opioids** |  |  |  |  |
| Buprenorphine (N02AE01) | Transdermal | 110 | 1.2 |  |
| Fentanyl (N02AB03) | Transdermal | 100 | 1.2 |  |
| Hydromorphone (N02AA03) | Oral | 20 | 3.6 |  |
| Ketobemidone (N02AB01) | Oral | 50 | 1 |  |
| Ketobemidone with spasmolytics (N02AG02) | Oral | 5 | 2 |  |
| Morphine; morphine combinations (N02AA01; N02AG01) | Oral | 100 | 1 | N02AG01 only available in Norway and Sweden |
| Nicomorphine (N02AA04) | Oral | 30 | 1* | Available only in Denmark |
| Oxycodone; oxycodone and naloxone (N02AA05; N02AA55) | Oral | 75 | 1.6 | Data on N02AA55 not available in Finland  Annual prevalence of N02AA55 reported separately in Denmark |
| Pethidine (N02AB02) | Parenteral | 400 | 0.3 |  |
| Pentazocine (N02AD01) | Oral | 200 | 0.17 |  |
| Tapentadol (N02AX06) | Oral | 400 | 0.1 |  |

*Conversion factor from Nissen et al. 2019^4^. ATC = Anatomical therapeutic chemical; DDD = Defined Daily Dose. DDD values and conversion factors are displayed for the most frequent forms of administration.

| Supplemental Table 2. Values in Figure 1: Annual prevalence (%) of opioid utilisation among Nordic residents aged ≥65. | | | | | | | | | | |
| --- | --- | --- | --- | --- | --- | --- | --- | --- | --- | --- |
| Country | 2009 | 2010 | 2011 | 2012 | 2013 | 2014 | 2015 | 2016 | 2017 | 2018 |
| Denmark | 18.55 | 18.95 | 18.82 | 18.65 | 18.56 | 18.76 | 18.54 | 18.47 | 17.94 | 16.76 |
| Finland | 13.31 | 13.46 | 13.73 | 13.89 | 13.89 | 13.62 | 13.75 | 13.66 | 13.54 | 13.20 |
| Iceland | 30.20 | 30.62 | 30.54 | 31.19 | 31.80 | 31.31 | 32.68 | 33.36 | 33.07 | 31.72 |
| Norway | 18.73 | 18.75 | 18.64 | 18.59 | 18.68 | 18.81 | 18.99 | 18.95 | 18.68 | 18.34 |
| Sweden* | 17.98 | 17.73 | 17.46 | 17.16 | 16.98 | 16.71 | 16.59 | 16.32 | 15.88 |  |

*Data available 2009-2017.

| Supplemental Table 3. Values in Figure 2: Opioid utilisation in Defined Daily Doses (DDD)s/1000 inhabitant/day. | | | | | | | | | | |
| --- | --- | --- | --- | --- | --- | --- | --- | --- | --- | --- |
| Country | 2009 | 2010 | 2011 | 2012 | 2013 | 2014 | 2015 | 2016 | 2017 | 2018 |
| Denmark | 58.50 | 59.11 | 58.66 | 58.36 | 57.12 | 58.10 | 57.06 | 56.20 | 53.39 | 47.88 |
| Finland | 28.34 | 28.39 | 28.41 | 28.33 | 27.70 | 26.98 | 26.32 | 25.63 | 24.55 | 23.05 |
| Iceland | 56.15 | 60.77 | 60.61 | 61.57 | 63.40 | 62.38 | 62.31 | 64.70 | 62.20 | 54.57 |
| Norway | 38.58 | 37.78 | 37.03 | 36.03 | 35.90 | 36.21 | 35.90 | 35.78 | 34.64 | 33.34 |
| Sweden* | 42.93 | 41.65 | 39.44 | 37.69 | 36.53 | 34.86 | 33.51 | 31.59 | 29.19 |  |

*Data available 2009-2017.

| Supplemental Table 4. Values in Figure 3: Opioid utilisation in morphine milligram equivalents (MME)s/user/day. | | | | | | | | | | |
| --- | --- | --- | --- | --- | --- | --- | --- | --- | --- | --- |
| Country | 2009 | 2010 | 2011 | 2012 | 2013 | 2014 | 2015 | 2016 | 2017 | 2018 |
| Denmark | 19.58 | 19.55 | 19.60 | 19.92 | 19.51 | 19.36 | 19.21 | 19.00 | 18.97 | 19.01 |
| Finland | 6.45 | 6.99 | 7.30 | 7.64 | 8.13 | 8.66 | 8.96 | 9.41 | 9.63 | 9.87 |
| Iceland | 4.42 | 4.99 | 4.92 | 5.38 | 5.56 | 5.69 | 5.29 | 5.45 | 5.18 | 4.55 |
| Norway | 6.20 | 6.53 | 6.89 | 7.11 | 7.48 | 7.76 | 7.89 | 8.27 | 8.52 | 8.52 |
| Sweden* | 9.63 | 10.07 | 10.43 | 10.53 | 10.96 | 11.18 | 11.32 | 11.20 | 10.91 |  |

*Data available 2009-2017.

| Supplemental Table 5. Values in Figure 4: Annual prevalence (%) of the most frequent opioids among Nordic residents aged ≥65. | | | | | | | | | | | |  |
| --- | --- | --- | --- | --- | --- | --- | --- | --- | --- | --- | --- | --- |
| Opioid | Country | 2009 | 2010 | 2011 | 2012 | 2013 | 2014 | 2015 | 2016 | 2017 | 2018 | |
| Buprenorphine | Denmark | 1.95 | 1.85 | 1.74 | 1.67 | 1.39 | 1.25 | 1.21 | 1.18 | 1.15 | 1.04 | |
|  | Finland | 0.75 | 1.55 | 2.20 | 2.51 | 2.84 | 3.01 | 3.17 | 3.23 | 3.33 | 3.41 | |
|  | Iceland | 0.58 | 0.58 | 0.60 | 0.71 | 0.79 | 0.74 | 0.78 | 0.95 | 0.97 | 0.88 | |
|  | Norway | 1.07 | 1.19 | 1.23 | 1.33 | 1.35 | 1.39 | 1.46 | 1.44 | 1.42 | 1.36 | |
|  | Sweden* | 1.06 | 1.36 | 1.71 | 1.82 | 1.95 | 1.97 | 1.90 | 1.82 | 1.67 |  | |
| Codeine | Denmark | 2.26 | 2.24 | 1.98 | 1.73 | 1.85 | 2.20 | 2.06 | 1.90 | 1.75 | 1.60 | |
|  | Finland | 9.47 | 9.19 | 9.06 | 8.92 | 8.51 | 7.96 | 7.70 | 7.31 | 6.90 | 6.28 | |
|  | Iceland | 24.94 | 24.76 | 24.44 | 24.99 | 25.69 | 25.40 | 26.86 | 27.21 | 27.08 | 25.94 | |
|  | Norway | 13.85 | 13.49 | 13.14 | 12.63 | 12.19 | 11.76 | 11.44 | 11.04 | 10.54 | 10.11 | |
|  | Sweden | 4.47 | 4.90 | 5.56 | 5.68 | 5.58 | 5.21 | 4.94 | 4.51 | 4.11 |  | |
| Fentanyl | Denmark | 1.58 | 1.53 | 1.42 | 1.39 | 1.48 | 1.47 | 1.45 | 1.43 | 1.34 | 1.21 | |
|  | Finland | 0.78 | 0.72 | 0.66 | 0.67 | 0.69 | 0.72 | 0.73 | 0.74 | 0.73 | 0.69 | |
|  | Iceland | 0.53 | 0.66 | 0.68 | 0.72 | 0.79 | 0.76 | 0.79 | 0.78 | 0.76 | 0.61 | |
|  | Norway | 0.50 | 0.52 | 0.52 | 0.54 | 0.56 | 0.57 | 0.58 | 0.58 | 0.57 | 0.55 | |
|  | Sweden | 0.95 | 1.00 | 1.01 | 0.94 | 0.93 | 0.91 | 0.91 | 0.84 | 0.77 |  | |
| Morphine | Denmark | 1.56 | 1.93 | 2.42 | 2.59 | 2.61 | 2.53 | 2.43 | 2.40 | 2.66 | 2.77 | |
|  | Finland | 0.03 | 0.03 | 0.03 | 0.03 | 0.04 | 0.05 | 0.05 | 0.06 | 0.06 | 0.06 | |
|  | Iceland | 0.85 | 1.08 | 1.24 | 1.40 | 1.40 | 1.34 | 1.23 | 1.17 | 1.01 | 0.98 | |
|  | Norway | 0.15 | 0.15 | 0.15 | 0.15 | 0.15 | 0.15 | 0.15 | 0.16 | 0.16 | 0.16 | |
|  | Sweden | 1.70 | 2.16 | 2.64 | 3.15 | 3.04 | 2.82 | 2.70 | 2.50 | 2.30 |  | |
| Oxycodone | Denmark | 2.01 | 1.92 | 1.30 | 1.02 | 1.01 | 1.11 | 1.25 | 1.45 | 1.59 | 1.84 | |
|  | Finland | 0.88 | 0.97 | 1.02 | 1.10 | 1.35 | 1.61 | 1.99 | 2.31 | 2.58 | 2.81 | |
|  | Iceland | 0.28 | 0.32 | 0.37 | 0.69 | 0.82 | 1.17 | 1.50 | 1.90 | 2.04 | 2.16 | |
|  | Norway | 0.56 | 0.65 | 0.70 | 0.79 | 0.93 | 1.04 | 1.15 | 1.30 | 1.49 | 1.58 | |
|  | Sweden | 3.04 | 3.41 | 3.71 | 4.05 | 5.04 | 5.99 | 6.86 | 7.50 | 7.95 |  | |
| Tramadol | Denmark | 11.45 | 11.84 | 12.16 | 12.34 | 12.10 | 11.90 | 11.69 | 11.40 | 10.22 | 8.24 | |
|  | Finland | 3.46 | 3.29 | 3.18 | 3.11 | 2.96 | 2.83 | 2.77 | 2.65 | 2.58 | 2.44 | |
|  | Iceland | 8.04 | 8.72 | 8.70 | 8.45 | 7.98 | 7.69 | 7.89 | 8.00 | 7.78 | 6.94 | |
|  | Norway | 5.27 | 5.75 | 6.01 | 6.38 | 6.75 | 7.22 | 7.58 | 7.72 | 7.54 | 7.30 | |
|  | Sweden | 6.74 | 6.77 | 6.83 | 5.66 | 4.48 | 3.62 | 3.06 | 2.55 | 2.10 |  | |

*Data available 2009-2017.

Supplemental Table 6. Values in Figure 5: Annual prevalence (%) of opioid utilisation according to gender.

| Country | Gender | 2009 | 2010 | 2011 | 2012 | 2013 | 2014 | 2015 | 2016 | 2017 | 2018 |
| --- | --- | --- | --- | --- | --- | --- | --- | --- | --- | --- | --- |
| Denmark | Men | 14.98 | 15.34 | 15.40 | 15.29 | 15.33 | 15.54 | 15.45 | 15.55 | 15.15 | 14.15 |
|  | Women | 21.35 | 21.82 | 21.58 | 21.40 | 21.22 | 21.43 | 21.12 | 20.92 | 20.31 | 18.97 |
| Finland | Men | 12.08 | 12.06 | 12.22 | 12.36 | 12.30 | 12.01 | 12.13 | 12.11 | 11.98 | 11.64 |
|  | Women | 14.18 | 14.45 | 14.83 | 15.01 | 15.07 | 14.84 | 15.00 | 14.86 | 14.75 | 14.42 |
| Iceland | Men | 26.14 | 26.26 | 25.98 | 26.69 | 27.50 | 27.46 | 28.54 | 29.27 | 28.58 | 27.62 |
|  | Women | 33.64 | 34.33 | 34.48 | 35.13 | 35.57 | 34.71 | 36.41 | 37.04 | 37.16 | 35.47 |
| Norway | Men | 15.90 | 15.91 | 15.96 | 15.87 | 15.99 | 16.18 | 16.41 | 16.39 | 16.11 | 15.93 |
|  | Women | 20.93 | 20.98 | 20.77 | 20.78 | 20.89 | 20.99 | 21.15 | 21.12 | 20.88 | 20.42 |
| Sweden* | Men | 15.29 | 15.06 | 14.98 | 14.68 | 14.61 | 14.38 | 14.34 | 14.16 | 13.85 |  |
|  | Women | 20.11 | 19.88 | 19.49 | 19.19 | 18.95 | 18.68 | 18.50 | 18.16 | 17.62 |  |

*Data available 2009-2017.

| Supplemental Table 7. Values in Figure 6: Annual prevalence (%) of opioid utilisation according to age group. | | | | | | | | | | | |
| --- | --- | --- | --- | --- | --- | --- | --- | --- | --- | --- | --- |
| Country | Age group (years) | 2009 | 2010 | 2011 | 2012 | 2013 | 2014 | 2015 | 2016 | 2017 | 2018 |
| Denmark | 65-69 | 12.95 | 13.31 | 13.25 | 13.03 | 13.07 | 13.47 | 13.35 | 13.27 | 12.87 | 11.94 |
|  | 70-74 | 15.94 | 16.57 | 16.43 | 16.39 | 16.27 | 16.54 | 16.21 | 16.06 | 15.32 | 14.04 |
|  | 75-79 | 19.56 | 20.26 | 20.31 | 20.37 | 20.09 | 20.24 | 19.89 | 19.66 | 19.00 | 17.29 |
|  | 80-84 | 23.82 | 24.00 | 24.11 | 24.11 | 24.30 | 24.23 | 24.16 | 24.06 | 23.33 | 21.94 |
|  | 85-89 | 28.83 | 29.37 | 29.42 | 29.54 | 29.31 | 29.17 | 28.65 | 28.83 | 28.27 | 27.06 |
|  | 90+ | 34.98 | 36.38 | 36.49 | 36.46 | 36.93 | 36.94 | 36.59 | 36.54 | 36.31 | 35.56 |
| Finland | 65-69 | 10.86 | 10.57 | 10.59 | 10.56 | 10.40 | 9.95 | 10.02 | 9.73 | 9.54 | 9.04 |
|  | 70-74 | 12.71 | 12.75 | 12.64 | 12.75 | 12.39 | 11.91 | 11.62 | 11.25 | 11.02 | 10.46 |
|  | 75-79 | 14.54 | 14.52 | 14.88 | 14.93 | 15.01 | 14.44 | 14.38 | 14.12 | 13.74 | 13.22 |
|  | 80-84 | 15.45 | 15.97 | 16.46 | 16.93 | 17.39 | 17.38 | 17.53 | 17.57 | 17.35 | 16.99 |
|  | 85-89 | 15.77 | 16.90 | 18.30 | 19.08 | 19.61 | 20.35 | 21.45 | 21.72 | 22.33 | 22.75 |
|  | 90+ | 15.31 | 17.77 | 19.68 | 21.46 | 22.97 | 24.74 | 26.74 | 28.50 | 29.61 | 30.69 |
| Iceland | 65-69 | 29.77 | 28.91 | 28.49 | 29.83 | 29.74 | 29.69 | 31.21 | 32.00 | 31.42 | 30.33 |
|  | 70-74 | 31.01 | 30.74 | 29.37 | 29.82 | 30.61 | 30.05 | 31.55 | 31.92 | 31.47 | 30.03 |
|  | 75-79 | 32.40 | 33.07 | 33.41 | 33.63 | 33.44 | 32.84 | 33.73 | 34.76 | 33.88 | 32.13 |
|  | 80-84 | 31.16 | 32.02 | 32.85 | 33.41 | 35.10 | 34.28 | 35.98 | 35.83 | 37.59 | 35.70 |
|  | 85-89 | 26.85 | 30.15 | 30.82 | 31.72 | 34.07 | 32.81 | 34.38 | 36.01 | 36.15 | 35.87 |
|  | 90+ | 20.73 | 26.44 | 30.06 | 29.01 | 30.99 | 31.41 | 32.44 | 33.97 | 32.80 | 31.67 |
| Norway | 65-69 | 16.04 | 16.04 | 15.95 | 16.08 | 16.42 | 16.49 | 16.70 | 16.65 | 16.37 | 16.14 |
|  | 70-74 | 18.08 | 18.09 | 17.99 | 17.69 | 17.61 | 17.76 | 17.80 | 17.85 | 17.59 | 17.39 |
|  | 75-79 | 20.26 | 20.18 | 20.10 | 20.09 | 20.04 | 20.16 | 20.39 | 20.23 | 19.70 | 19.08 |
|  | 80-84 | 21.28 | 21.39 | 21.51 | 21.49 | 21.56 | 21.85 | 22.01 | 21.73 | 21.53 | 21.07 |
|  | 85-89 | 21.46 | 21.80 | 21.66 | 21.73 | 21.85 | 22.36 | 22.53 | 22.63 | 22.57 | 21.94 |
|  | 90+ | 17.58 | 18.41 | 18.90 | 19.31 | 19.73 | 19.67 | 20.39 | 20.72 | 20.76 | 20.63 |
| Sweden* | 65-69 | 13.20 | 13.13 | 12.90 | 12.45 | 12.14 | 11.75 | 11.57 | 11.32 | 11.16 |  |
|  | 70-74 | 15.34 | 15.07 | 14.89 | 14.85 | 14.80 | 14.51 | 14.47 | 14.11 | 13.37 |  |
|  | 75-79 | 17.79 | 17.54 | 17.48 | 17.29 | 17.47 | 17.30 | 17.06 | 16.77 | 16.49 |  |
|  | 80-84 | 21.39 | 21.17 | 20.94 | 20.79 | 20.31 | 20.28 | 20.18 | 19.97 | 19.35 |  |
|  | 85-89 | 27.36 | 26.11 | 25.66 | 25.57 | 25.56 | 25.25 | 25.20 | 24.81 | 24.10 |  |
|  | 90+ | 29.65 | 32.47 | 32.47 | 32.10 | 32.83 | 33.35 | 33.63 | 33.54 | 32.95 |  |

*Data available 2009-2017.

Supplemental Table 8. Values in Supplemental Figure 2: Annual prevalence (%) of buprenorphine according to gender

| Country | Gender | 2009 | 2010 | 2011 | 2012 | 2013 | 2014 | 2015 | 2016 | 2017 | 2018 |
| --- | --- | --- | --- | --- | --- | --- | --- | --- | --- | --- | --- |
| Denmark | Men | 1.16 | 1.07 | 1.02 | 0.97 | 0.80 | 0.73 | 0.70 | 0.72 | 0.71 | 0.64 |
|  | Women | 2.57 | 2.48 | 2.33 | 2.24 | 1.89 | 1.68 | 1.63 | 1.57 | 1.53 | 1.38 |
| Finland | Men | 0.47 | 0.91 | 1.30 | 1.45 | 1.65 | 1.75 | 1.87 | 1.92 | 2.00 | 2.06 |
|  | Women | 0.95 | 2.01 | 2.85 | 3.29 | 3.73 | 3.97 | 4.17 | 4.24 | 4.37 | 4.47 |
| Iceland | Men | 0.22 | 0.23 | 0.26 | 0.31 | 0.38 | 0.41 | 0.39 | 0.47 | 0.45 | 0.42 |
|  | Women | 0.89 | 0.88 | 0.88 | 1.07 | 1.15 | 1.04 | 1.14 | 1.37 | 1.44 | 1.29 |
| Norway | Men | 0.61 | 0.64 | 0.66 | 0.70 | 0.70 | 0.73 | 0.77 | 0.75 | 0.75 | 0.72 |
|  | Women | 1.43 | 1.62 | 1.69 | 1.85 | 1.89 | 1.94 | 2.04 | 2.03 | 2.00 | 1.92 |
| Sweden* | Men | 0.64 | 0.80 | 1.00 | 1.07 | 1.14 | 1.15 | 1.11 | 1.08 | 1.00 |  |
|  | Women | 1.39 | 1.80 | 2.28 | 2.43 | 2.63 | 2.65 | 2.56 | 2.45 | 2.25 |  |

*Data available 2009-2017.

**References**

1. WHO Collaborating Center for Drug Statistics Methodology. Norwegian Institute of Public Health. The Anatomical Therapeutic Chemical Classification System. http://www.whocc.no/atc_ddd_index/ (2020).

2. Sundhedsdatastyrelsen. Medstat. www.medstat.dk (2020).

3. Norwegian Health Economics Administration. Morphine equivalency ratios. http://www.helfoweb.com/morfinekvivalenter/ (2020).

4. Nissen, S. K., Pottegård, A. & Ryg, J. Trends of Opioid Utilisation in Denmark: A Nationwide Study. *Drugs - Real World Outcomes* **6**, 155–164 (2019).
